# Determination of the iron(IV) local spin states of the Q intermediate of soluble methane monooxygenase by Kβ X-ray emission spectroscopy

**DOI:** 10.1007/s00775-022-01953-4

**Published:** 2022-08-21

**Authors:** George E. Cutsail, Rahul Banerjee, Derek B. Rice, Olivia McCubbin Stepanic, John D. Lipscomb, Serena DeBeer

**Affiliations:** 1 Max Planck Institute for Chemical Energy Conversion, Stiftstrasse 34-36, 45470 Mülheim an der Ruhr, Germany; 2 Institute of Inorganic Chemistry, University of Duisburg-Essen, Universitätsstrasse 5-7, 45117 Essen, Germany; 3 Department of Biochemistry Molecular Biology and Biophysics, University of Minnesota, Minneapolis, MN 55455 USA

**Keywords:** Methane monooxygenase, Non-heme iron, Spin state, X-ray emission spectroscopy, Rapid freeze-quench

## Abstract

Soluble methane monooxygenase (sMMO) facilitates the conversion of methane to methanol at a non-heme Fe^IV^_2_ intermediate MMOH_Q_, which is formed in the active site of the sMMO hydroxylase component (MMOH) during the catalytic cycle. Other biological systems also employ high-valent Fe^IV^ sites in catalysis; however, MMOH_Q_ is unique as Nature’s only identified Fe^IV^_2_ intermediate. Previous ^57^Fe Mössbauer spectroscopic studies have shown that MMOH_Q_ employs antiferromagnetic coupling of the two Fe^IV^ sites to yield a diamagnetic cluster. Unfortunately, this lack of net spin prevents the determination of the local spin state (*S*_loc_) of each of the irons by most spectroscopic techniques. Here, we use Fe Kβ X-ray emission spectroscopy (XES) to characterize the local spin states of the key intermediates of the sMMO catalytic cycle, including MMOH_Q_ trapped by rapid-freeze-quench techniques. A pure XES spectrum of MMOH_Q_ is obtained by subtraction of the contributions from other reaction cycle intermediates with the aid of Mössbauer quantification. Comparisons of the MMOH_Q_ spectrum with those of known *S*_loc_ = 1 and *S*_loc_ = 2 Fe^IV^ sites in chemical and biological models reveal that MMOH_Q_ possesses *S*_loc_ = 2 iron sites. This experimental determination of the local spin state will help guide future computational and mechanistic studies of sMMO catalysis.

**Graphical abstract:**

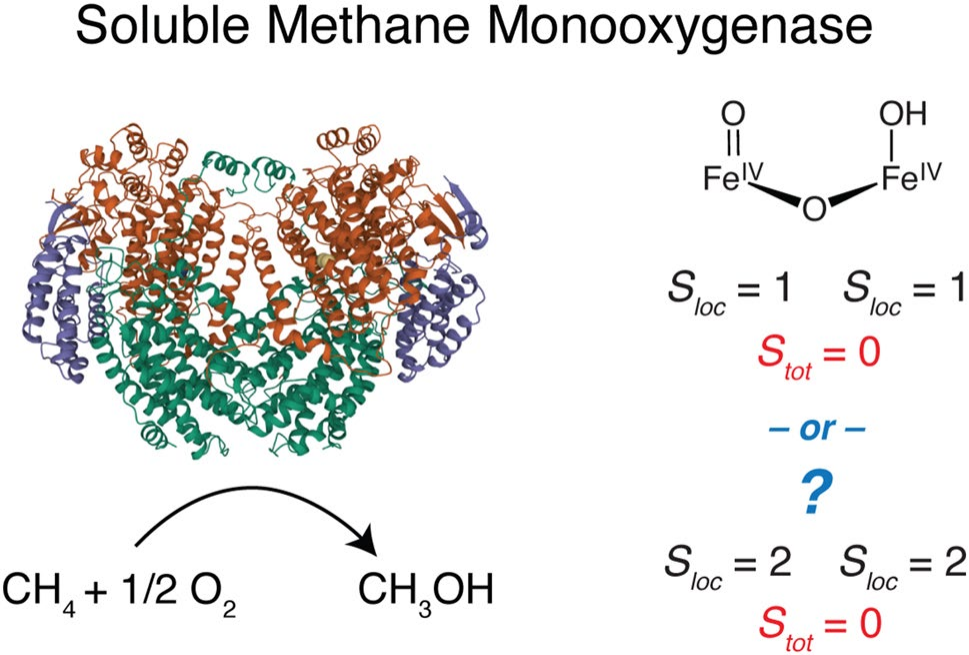

**Supplementary Information:**

The online version contains supplementary material available at 10.1007/s00775-022-01953-4.

## Introduction

The conversion of methane to methanol is of significant interest for technologies aimed at gas-to-liquid fuel conversion, and for the sequestration of a greenhouse gas, alongside the desire to understand the fundamental chemistry of strong C–H bond activation. Nature facilitates this challenging chemical reaction with two different methane monooxygenase enzymes [1]. The most common type is the copper-containing particulate methane monooxygenase (pMMO) [2–4], which is a topic of intense research, in particular for the assignment of the active site and its structure [5–11]. In copper limited environments [2, 12], methanotrophic bacteria express the iron-containing soluble methane monooxygenase (sMMO), which has a well characterized non-heme carboxylate-bridged di-iron active site [13, 14].

**Fig. 1:**
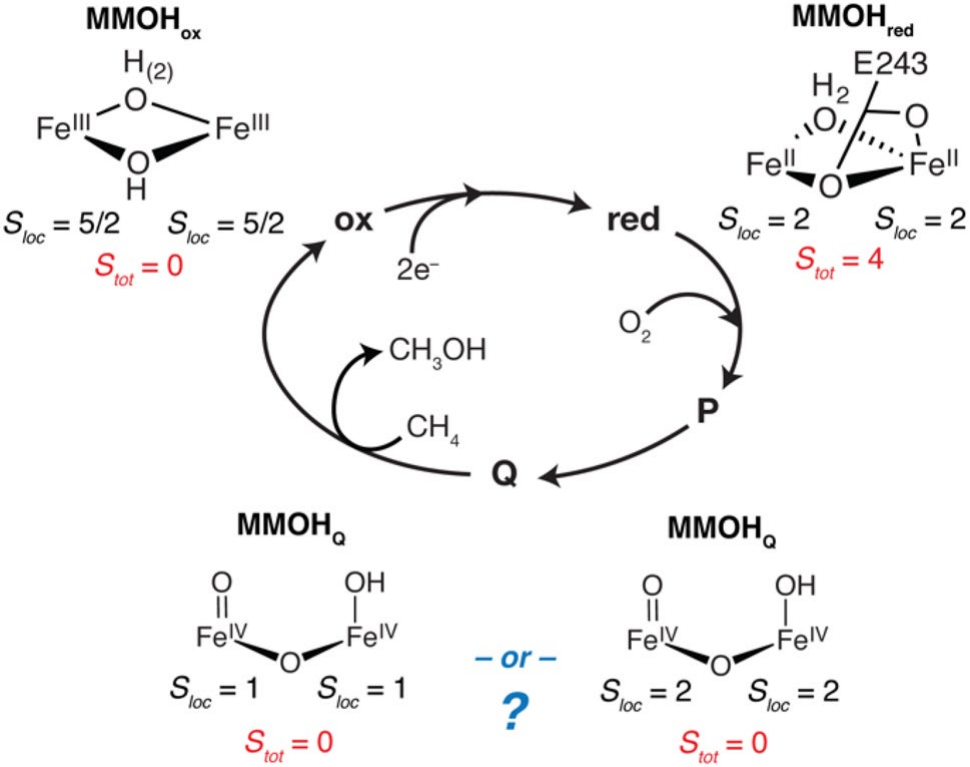
Abbreviated catalytic scheme of sMMO with corresponding iron oxidation states, local spin states (*S*_loc_) and cluster ground spin state (*S*_tot_) for MMOH_ox_, MMOH_red_, and MMOH_Q_. The structure of MMOH_Q_ is drawn as the proposed ‘open-core’ structure [23, 24], although a closed Fe_2_(µ-O)_2_ core is also proposed [21, 26]

The active site of sMMO is located within the hydroxylase protein (MMOH) and forms various catalytic intermediates that have been spectroscopically identified [13, 15–25]. An abbreviated catalytic mechanism of MMOH is shown in Fig. 1. For the critical intermediate of interest, MMOH_Q_, the O–O bond of the preceding peroxo intermediate (MMOH_P_) has been cleaved and the resultant intermediate is able to activate the strong 105 kcal/mol C–H bond of methane, allowing for its subsequent conversion to methanol. To our knowledge, MMOH_Q_ is the only di-iron(IV) intermediate that has been identified in Nature [17, 19, 20].

The exact geometric structure of the MMOH_Q_ intermediate has been hotly debated for over two decades [14, 17, 18, 21, 23–27]. Spectroscopic characterizations of MMOH_Q_ have included vibrational studies (transient resonant Raman and nuclear resonance vibrational scattering, NRVS), which have also been used to argue for a “closed-core” bis-µ-oxo structure [21, 25]. However, high-resolution Fe K-edge X-ray absorption (HERFD XAS) characterization and pre-edge analysis of MMOH_Q_ in comparison with biomimetic models support an “open core” structure [23]. Recent HERFD extended X-ray absorption fine structure (EXAFS) measurements of RFQ samples of sMMO were fit with a long Fe–Fe distance of ~ 3.3 Å and showed no evidence for the initially reported 2.46 Å di-iron distance that would have supported a closed-core structure [24]. The short Fe–Fe distance in the previous 1997 EXAFS report [18] was shown to arise from metallic iron background scattering contributions, which are suppressed in the HERFD EXAFS [24]. Because the RFQ samples in the HERFD EXAFS study contained primarily MMOH_Q_ (~ 50%) and MMOH_red_ (40%), the fitted distance is weighted average of these contributions. As the di-iron distance in MMOH_red_ is established to be ~ 3.2 Å [28], the longer fitted distance of the RFQ samples therefore suggests a longer di-iron distance for MMOH_Q_ of ~ 3.4 Å, consistent with an open core structure [24]. Further, a recent quantum mechanics-molecular mechanics (QM/MM) computational study of MMOH_Q_ has shown that the computed spectroscopic signatures of an open core structure are consistent with the Mössbauer, Fe HERFD pre-edge XAS, EXAFS di-iron distance measurements, and resonance Raman [29]. These QM/MM computational studies, however, have not yet been extended to the recently reported NRVS spectrum of MMOH_Q_ [25].

While the geometric structure of MMOH_Q_ remains controversial, its electronic structure is also key to understanding catalytic activity and predicting spectroscopic signatures. The initial spectroscopic characterization and identification of MMOH_Q_ included its characteristic optical absorption at 430 nm and a single observed Mössbauer quadruple doublet in the case of MMOH_Q_ trapped using the enzyme from *Methylosinus trichosporium* OB3b [16, 19, 20]. The single Mössbauer doublet from two irons was interpreted to indicate that the iron sites have similar coordination environments, thereby yielding overlapping doublets. The isomer shift of the observed doublet is entirely consistent with Fe^IV^ oxidation state, however, the Mössbauer characterization of MMOH_Q_ remains inconclusive for the assigned local spin states of the iron sites. Low temperature and applied magnetic field Mössbauer spectroscopy of MMOH_Q_ determined that the total ground spin state of the cluster is *S*_tot_ = 0, meaning that the Fe^IV^ ions are antiferromagnetically coupled, where iron sites must have local spin states (*S*_loc_) of intermediate-spin (*S*_loc_ = 1) or high-spin (*S*_loc_ = 2) [20]. As originally stated by Münck, the isomer shift and quadrupole splittings of MMOH_Q_ are consistent with both *S*_loc_ = 1 and *S*_loc_ = 2 local spin states; the applied field measurement only serves to measure the coupling strength between the two sites [20]. Non-diamagnetic cluster spin states allow for clear local spin-state assignments by Mössbauer spectroscopy. For other mononuclear Fe^IV^ synthetic chemical and biological centers, applied field Mössbauer measurement has been essential to differentiate between intermediate and high-spin iron [30–34]. For example, in the case of the *S*_tot_ = 1/2 Fe^III^–Fe^IV^ cluster intermediate (X) of ribonucleotide reductase (RNR) [35], the paramagnetic nature of the cluster allowed iron hyperfine and local spin states to be determined by applied field measurements.

Attempts to understand or model both the local and cluster spin states of MMOH_Q_ through biomimetic chemistry have also been challenging [33, 36–39]. Different local and cluster spin states have been identified in these models, including those with *S*_loc_ = 1 or 2 and ferro- or antiferromagnetic coupling of the iron sites. A sampling of both mono and di-nuclear biomimetic models and their Mössbauer parameters are listed in Table 1. The observed Mössbauer isomer shifts and quadrupole splittings for both *S*_loc_ = 1 and 2 Fe^IV^ sites span a fairly wide range around what is observed for MMOH_Q_, making a direct assignment of a local spin state from these parameters alone prohibitive.

**Table 1:** Mössbauer parameters of various Fe^IV^ sites

	*S*_loc_	*S*_tot_	*δ* (mm/s)	Δ*E*_*Q*_ (mm/s)	References
MMOH_Q_		0	0.17	0.53	[20]
TauD J	2	2	0.31	− 0.88	[40]
RnR X (Fe^III^-**Fe**^**IV**^) ^a^	5/2, 2	1/2	0.26	− 0.6	[35]
[L(OH)Fe-(µ-O)**Fe(O)**L]^3+^; L = TPA* ^a,b^	1, 1	0	− 0.03	+ 0.92	[41]
[L**(OH)Fe**-(µ-O)Fe(O)L]^3+^; L = TPA* ^a,b^	1, 1	0	0.0	+ 1.96	[41]
[Fe^IV^_2_(μ-O)_2_(TPA*)_2_]^4+^	1, 1	0	− 0.04	2.09	[36]
[(O)(L)Fe^IV^−O−Fe^IV^(O)(L)]^2+^; L = TPA* ^b^	2, 2	0	0.14	0.52	[39]
[(O)(L)Fe^IV^−O−Fe^IV^(O)(L)]^2+^; L = TPA* ^b^	1, 2	3	− 0.02, 0.14	− 1.17, 0.82	[39]
[(TMC)Fe(O)NCMe]^2+ c^	1	1	0.17	+ 1.24	[42]
[(N4Py)Fe(O)]^2+ d^	1	1	− 0.04	0.93	[43]
[Fe^IV^(O)(2PyN2Q)]^2+ e^	1	1	0.03	0.56	[44]
[Fe^IV^(O)(TMG_3_tren)]^2+ f^	2	2	0.24	− 0.29	[45]
[Fe^IV^(O)(*t*Bu_3_TACN)]^2+ g^	2	2	0.11	+ 0.96	[46]
Aqueous Fe^IV^ = O	2	2	0.38	− 0.33	[47]

^a^In the case of asymmetric di-iron cluster, the parameters are detailed for the iron in bold

^b^TPA* = tris(4-methoxy-3,5-dimethylpyridyl-2-methyl)amine

^c^TMC = 1,4,8,11-tetramethyl-1,4,8,11-tetraazacyclotetradecane

^d^N4Py = *N*,*N*-bis(2-pyridylmethyl)bis(2pyridyl)methylamine

^e^2PyN2Q = 1,1-di(pyridin-2-yl)-*N*,*N*-bis(quinolin-2-ylmethyl)methanamine)

^f^TMG_3_tren = tris[2-{N-tetramethylguanidyl}ethyl]amine]

^g^*t*Bu_3_TACN = 1,4,7-tri-tert-butyl-1,4,7-triazacyclononane

Perhaps the primary support for the now more commonly accepted *S*_loc_ = 2 spin state of MMOH_Q_ arises in part from analogy to other non-heme iron chemistry [39]. Enzymatic non-heme Fe^IV^ sites favor local high-spin states, in part due to their weak-field ligand environments [30], similar to that present in MMOH. Furthermore, the enhanced reactivity of high-spin *S* = 2 Fe^IV^ sites to perform hydrogen atom transfer reactions, as seen in other forms of non-heme biological catalysis and biomimetic chemistry [31, 33, 34, 38, 48, 49], is often used to infer a spin-state assignment for MMOH_Q_. Cryo-reduction experiments of MMOH_Q_ have also revealed an electronic structure similar in character to RNR-X, suggesting locally high-spin Fe^IV^ sites [50]. Previous computational studies of MMOH_Q_ also favor *S*_loc_ = 2 iron spin states [29, 51–53]. Ultimately, these chemical analogies or other studies have fallen short of a direct experimental characterization of the local spin states in MMOH_Q_, motivating an investigation of the electronic structure by means of an alternative spectroscopic approach.

For 3d transition metals, Kβ (3p → 1s) X-ray emission spectroscopy (XES) has been well demonstrated to be excellent reporter of electronic structure due to 3p–3d exchange in the final state [54–58]. Kβ mainlines have previously been demonstrated to both report and correlate to oxidation state and spin state. As a marker of oxidation state, the maximum of the Kβ mainline, the Kβ_1,3_ feature, generally shifts to higher energy with increased oxidation-state [54]. On the other hand, the intensity of the Kβ’ feature, at lower energy, correlates with the number of unpaired electrons, thereby allowing for spin-state assignments. For instance, for ferrous complexes, the presence or absence of the Kβ’ has been used as a marker of high-spin (*S* = 2) or low-spin (*S* = 0) electronic configurations [57–60].

While these rule-of-thumb trends may hold true in idealized systematic studies, covalency is shown to have profound influences on the shape and energies of Kβ mainlines [56, 58]. Increasing metal–ligand covalency delocalizes metal 3d orbital character onto the ligands and thus decreases the metal 3p-3d exchange integrals, which determine the final state splitting in Kβ spectra [56]. Ultimately, this relationship predicts that expected shifts of the Kβ_1,3_ to higher energy with increased oxidation-state may be counteracted by increased metal–ligand covalency, making some interpretations across an oxidation state series difficult. However, these challenges generally do not hinder the ability of Kβ XES to probe local spin states and distinguish between spin states in samples of known oxidation-state and similar covalency [54, 56–58, 61, 62]. Generally, Fe Kβ XES is not sensitive to the magnetic spin coupling (*J*) between the iron centers in bimetallic or larger clusters; therefore, the bulk XES measurement reports on the average local spin state of all iron sites [63–66]. This makes the approach attractive to possibly probe the local spin states in diamagnetic clusters such as MMOH_Q_ and distinguish between its local spin-state assignments.

## Experimental

The sMMO proteins ^57^Fe-enriched MMOH and MMOB were purified according to protocols described recently in the literature [22, 67]. The Fe Kβ mainline XES spectra were collected on frozen solution samples of MMOH_red_, MMOH_ox_ and freeze-quenched samples (MMOH-RFQ) to trap MMOH_Q_ of the same samples described in a previously published HERFD EXAFS study [24]. The MMOH-RFQ samples were prepared as previously described by freezing the reaction mixture in precooled sample cell of super-cooled liquid nitrogen (-199 °C) [24].

^57^Fe Mössbauer spectroscopy was used to quantitate the three components, MMOH_red_, MMOH_ox_ and MMOH_Q_, in the MMOH-RFQ sample. As previously reported [24], the frozen solution samples had 46% MMOH_Q_, 39.5% MMOH_red_, and 14.5% MMOH_ox_.

The *S* = 1, Fe^IV^ = O complex ([Fe^IV^(O)(2PyN2Q)](PF_6_)_2_ (2PyN2Q = 1,1-di(pyridin-2-yl)-*N*,*N*-bis(quinolin-2-ylmethyl)methanamine)) was synthesized and isolated following published procedures [44, 68]. Solid samples for XES data collection were prepared in 1.0 mm Al spacers and sealed with 38 µm thick Kapton tape.

The Fe Kβ XES data were collected at beam line ID-26 of the European Synchrotron Radiation Facility (ESRF) operating at 6 GeV and 200 mA. All samples were measured in a liquid helium cryostat operating at 20 K. The energy of the incident beam was selected using either a Si(111) or Si(311) double crystal monochromator, each of which was calibrated by setting the first inflection of an iron foil to 7111.2 eV. The incident monochromator was then set to an excitation energy of 7800 eV to non-resonantly excite the sample. The XES spectra were collected with a 1 m radius Johann spectrometer equipped with five Ge(620) spherically bent analyzer crystals and calibrated by the scanning of elastic scattering lines. A collected reference Kβ XES spectrum of Fe_2_O_3_ is displayed in Fig. S1. The measured resolution of the elastic scattering line was ~ 1.5 eV (fwhm) which includes the contribution of the upstream Si(111) monochromator. For the synthetic solid sample, an avalanche photodiode (APD) detector was used, while the more dilute protein samples required the use of an energy resolving Ketek detector to further improve the S/N of the emission spectra. For the XES collection of the protein, the incident beam was attenuated to a maximum estimated flux of 4 × 10^12^ photons/s within a spot size of 1.2 mm (*w*) × 0.1 mm (*v*) to further reduce radiation damage. Maximum sample exposure dwell times were determined through the evaluation of repeated fast X-ray near edge absorption (XANES) scans (5–60 s) to determine total acceptable photon doses [24]. Collection of the protein XES was completed in small segments (~ 12 eV wide, 50–61 points) of the entire XES spectra (7.025–7.08 keV) with 2 eV overlap per energy segment (10–11 data points). Each segment was collected on a fresh sample spot, limiting the total sample spot exposure time. The maximum dwell times per data point were: MMOH_red_, 1.25 secs; MMOH_ox_ 0.40 secs; MMOH_Q_, 0.15 secs. This limited the sample spot exposure times to a maximum of 76.25 secs (MMOH_red_), 24.4 secs (MMOH_ox_) and 15.25 secs (MMOH_Q_). This collection process allowed for improved signal-to-noise to be acquired at each data point, and to mitigate beam induced damage. All data segments were splined together through the averaging of their overlapping components. A sample of the process is shown in Fig. S2.

All Kβ XES spectra were normalized to a unit area of 1 over the energy range of 7025 to 7080 eV. The XES spectrum of pure MMOH_Q_ was calculated by subtracting the MMOH_red_ and MMOH_ox_ components from MMOH-RFQ at the ratios determined by Mössbauer and renormalizing to a unit area of 1. The first moments reported are determined over the described energy ranges by the following equation,

$${M}_{1}=\frac{{\sum }_{i}\left({E}_{i}{I}_{i}\right)}{{\sum }_{i}{I}_{i}}$$

where *E*_*i*_ and *I*_*i*_ are the energy and intensities, respectively, at data point *i*.

## Results

**Fig. 2:**
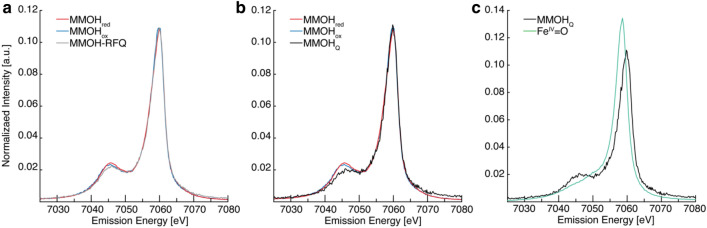
**a** Fe Kβ mainline XES of MMOH_red_, MMOH_ox_ and MMOH-RFQ samples of sMMO. **b** The MMOH_Q_ spectrum is determined by subtraction of the MMOH_red_ and MMOH_ox_ components from the MMOH-RFQ spectrum. **c** The MMOH_Q_ XES is compared to that of an *S* = 1 Fe^IV^ = O

Samples of MMOH_red_, MMOH_ox_ and rapid-freeze-quenched (RFQ) samples of MMOH_Q_ were prepared as previously described [24]. The Fe Kβ XES spectra of the three prepared samples of sMMO (MMOH_red/ox/RFQ_) are shown in Fig. 2a. No significant differences in the Kβ_1,3_ maxima are apparent. One does, however, observe slight intensity differences in the Kβ’ region (at ~ 7045 eV) following a trend of MMOH_red_ > MMOH_ox_ > MMOH-RFQ. As the RFQ sample is a mixture of all three different components and therefore three oxidation states, an assignment of the convoluted spectrum is challenging. The individual components of the RFQ samples were quantitated by ^57^Fe Mössbauer spectroscopy, with an estimated 46% yield of MMOH_Q_ [24]. The ^57^Fe Mössbauer quantification of each component in the MMOH-RFQ sample allows for the ‘pure’ MMOH_Q_ spectrum to be calculated as previously established in the HERFD XAS studies of sMMO and other non-heme di-iron proteins [23, 69].

The ‘pure’ MMOH_Q_ mainline spectrum is shown in Fig. 2b, overlaid with MMOH_red_ and MMOH_ox_. Here, one can clearly see that the Kβ_1,3_ peak position does not shift for the various oxidation states Fe^II^_2_/Fe^III^_2_/Fe^IV^_2_ of sMMO. Often, the first moment of the Kβ_1,3_ is used rather than the peak maximum to determine energy shifts because the first moment has been shown to have more sensitivity to small changes [54, 70–72]. However, the first moment analysis of the three spectra, Table 2, does not reveal significant energy shifts either, even though it was previously shown that all three of these samples exhibit clearly different XAS pre-edge and rising edge energies (see Figure S5 in Ref. [24]), consistent with their anticipated oxidation states and previous HERFD-XAS characterization [23].

**Table 2:** Key Fe Kβ mainline parameters

	Kβ First Moment^a^	Kβ_1,3_ First Moment^b^	Kβ’ First Moment^c^	Δ(Kβ_1,3_–Kβ’)
MMOH_red_	7054.94	7058.96	7043.59	15.37
MMOH_ox_	7055.00	7059.00	7043.75	15.25
RFQ	7055.20	7059.05	7043.59	15.46
MMOH_Q_	7055.45	7059.12	7043.43	15.69
Fe(IV) = O	7055.38	7058.16	7044.10	14.06

All values have energy units of eV

^a^Determined over entire measured Kβ mainline spectrum, 7025–7080 eV

^b^Determined over the energy range of 7051–7070 eV

^c^Determined over the energy range of 7025–7051 eV

While the Kβ_1,3_ may not exhibit clear differences with oxidation-state changes, the Kβ’ intensity of the three states clearly varies, Fig. 2b. The Kβ’ feature of MMOH_red_ appears slightly more intense than MMOH_ox_. This is in agreement with the subtle intensity differences observed in other high-spin Fe^II^ versus Fe^III^ mainlines, such as [Fe(H_2_O)_6_]^2+/3+^ [58]. MMOH_Q_ has a clear Kβ’ feature, indicating that there are unpaired d electrons (*S*_loc_ > 0). The first moment of the Kβ’ of MMOH_Q_ feature is at approximately the same energy as the more reduced iron species. In fact, the Kβ’ features for each of the three MMOH states appear at approximately the same energies, with only the intensities of the Kβ’ features varying, making the Δ(Kβ_1,3_– Kβ’) splitting approximately the same for each species, Table 2.

**Scheme 1:**
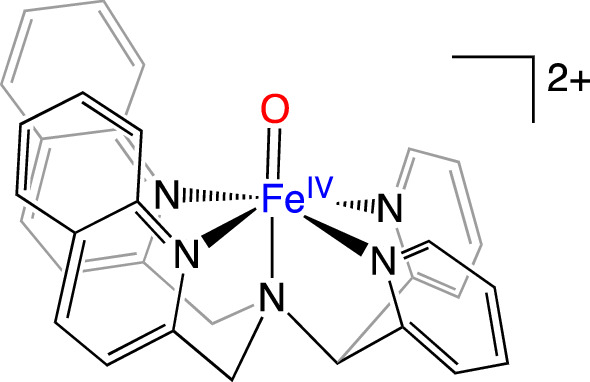
Fe^IV^ = O; [Fe(O)(2PyN2Q)]^2+^

The mainline of MMOH_Q_ may be the result of two local Fe^IV^ spin states: high-spin *S* = 2, or intermediate-spin *S* = 1. To determine the spin state, the mainline is directly compared to the Fe^IV^ *S* = 1 mainline from an Fe^IV^ = O complex [Fe(O)2PyN2Q)]^2+^, Scheme 1. Two immediate differences are observed in the spectra plotted in Fig. 2c. First, the Kβ_1,3_ energy for the Fe^IV^ = O *S* = 1 model complex is clearly shifted to lower energy by approximately 1 eV compared to MMOH_Q_ and second, the Kβ’ region for the Fe^IV^ = O model is significantly less intense than that of MMOH_Q_. Furthermore, the same first moment analysis of the Kβ’ of the Fe^IV^ = O mainline reveals an + 1 eV compared to MMOH_Q_, decreasing the Δ(Kβ_1,3_– Kβ’) splitting by ~ 2 eV, comparatively.

With respect to the Kβ’ intensity of MMOH_Q_, one may refer to the fingerprinting analyses performed for ferrous ion sites [57–60]. The presence of the Kβ’ in the high-spin *S* = 2 and its absence in low-spin S = 0 centers is used as a spin-state diagnostic tool. For MMOH_Q_, the clearly greater intensity of its Kβ’ feature and larger Δ(Kβ_1,3_– Kβ’) splitting compared to that of the established *S* = 1 Fe^IV^ = O in Fig. 2c strongly suggests *higher* local spin states for the Fe^IV^ ions of MMOH_Q_.

For the Δ(Kβ_1,3_– Kβ’) splitting, it has been previously demonstrated that the energetic splitting is proportional to the nominal spin of the metal center, but these energy shifts may be further modulated by covalency [56]. However, the quantification of the covalency in the various states of MMOH is difficult to estimate. One may naturally consider a potential di-iron(IV) open core structure that contains terminal oxos to be more covalent than the MMOH_ox_ and MMOH_red_ states. However, the previous Mn Kβ mainline characterization of the step-wise deprotonation of high-spin Mn^IV^ *S* = 2 Mn_2_(µ-OH)_2_ dimers to the Mn_2_(µ-O) cores exhibited only minimal shifts (~ 0.5 eV) in the Kβ_1,3_ energies [64]. This suggests that when comparing potential oxo vs hydroxo ligation to the iron sites, the covalency changes will result in only minor energetic perturbations and that local iron spin state should be the dominant contribution to the Kβ mainline energies. Our analysis and comparison of the sMMO Kβ mainline lines clearly supports the conclusion that the local spin states of the iron sites of MMOH_Q_ are *S* = 2. Furthermore, the Kβ mainline analysis of MMOH_Q_ as an *S*_loc_ = 2 is very consistent with the *S*_loc_ = 2 and *S*_loc_ = 5/2 spin states of the MMOH_red_ and MMOH_ox_, respectively [13, 73].

## Discussion

While previous Mössbauer experiments of MMOH_Q_ provide evidence for isomer shifts in clear agreement with Fe^IV^ ions, the antiferromagnetic coupling and *S*_tot_ = 0 cluster spin state do not yield conclusive information of the local spin states of the iron sites. Here, we have used Fe Kβ XES to probe the local spin state of MMOH_Q_. The ΔKβ splitting and the Kβ’ intensity of MMOH_Q_ are excellent indicators of local high-spin *S* = 2 iron sites that antiferromagnetically couple to form the *S*_tot_ = 0 cluster.

The Kβ mainline of MMOH_Q_ has distinct features that also resemble other assigned *S* = 2 Fe^IV^ centers [58, 74]. In a recent study of the Fe Kβ signatures of various compounds, the Kβ emission spectra of the iron perovskites LaFe^III^O_3_ and SrFe^IV^O_3_ were reported [58]. In LaFe^III^O_3_, a typical *S* = 5/2 ferric mainline is observed, whereas the high-spin *S* = 2 Fe^IV^ mainline of SrFe^IV^O_3_ exhibits a similar Kβ_1,3_ mainline maxima, but a slightly decreased Kβ’ intensity, similar to that observed for MMOH_Q_. However, we do note that this perovskite sample does not have the same molecular and/or electronic properties as the non-heme carboxylate-bridged di-iron center of sMMO, and so only qualitative comparisons are made.

Perhaps the most closely related structure to MMOH_Q_ is an RNR intermediate. The more widely studied di-iron RNR forms a high-valent Fe^III^–Fe^IV^ intermediate termed ‘X,’ that has a *S*_tot_ = 1/2 cluster spin state as described earlier. Similarly, the hetero-metallic Mn–Fe class I-c of RNRs also forms a high-valent intermediate Mn^IV^–Fe^IV^ intermediate [75]. For the d^4^ Fe center of the Mn^IV^–Fe^IV^ RNR, the Fe Kβ mainline does not exhibit any clear energy shifts of the Kβ_1,3_ for the local Fe^II^, Fe^III^ and Fe^IV^ centers, and the Fe^IV^ Kβ’ exhibits a decreased intensity relative to the lower Fe oxidation-state intermediates [74]. The Fe Kβ XES of the *S* = 2 Fe^IV^ center from Mn–Fe RNR has similar intensity to what we have observed for MMOH_Q_ (Figs. S3 and S4), lending additional support to the idea that measured Kβ XES of MMOH_Q_ reflects two *S* = 2 Fe^IV^ ions and not a beam damaged product yielding high-spin *S* = 5/2 ferric sites, which would further increase Kβ’ intensity and ΔKβ splitting.[25, 50] Furthermore, the ΔKβ splitting observed in RNR is similar to that seen here in MMOH_Q_, Fig. S4. It is important again to note the clear presence of a Kβ’ feature in the *S* = 2 Fe^IV^ RNR compared to the lack of a well-resolved Kβ’ in the *S* = 1 Fe^IV^ = O model reported here (Fig. S4), offering further support that that the Kβ mainline observed for MMOH_Q_ is diagnostic of an *S* = 2 local spin states at the iron atoms.

## Conclusion and outlook

In summary, the high-spin Fe^II^, Fe^III^ and Fe^IV^ sites of sMMO do not exhibit oxidation-state-dependent energy shifts by Fe Kβ XES. Modest differences are observed in the Kβ’ due to spin state. Notably, the ΔKβ(Kβ_1,3_-Kβ’) splitting of sMMO remains fairly constant, which is very consistent with high-spin states for MMOH_ox_, MMOH_red_, and MMOH_Q_. Direct comparison of the MMOH_Q_ mainline to a known *S* = 1 Fe^IV^ complex has shown dramatic spectral differences due to changes in the apparent spin state. This approach, in line with Fe Kβ XES spin state fingerprinting for ferrous and ferric iron, clearly distinguishes between the intermediate *S* = 1 spin of the model complex studied here and the apparent *S*_loc_ = 2 of MMOH_Q_. The Kβ XES experiment offers a clear experimental approach to determine local spin state in ambiguous systems, where magnetic spin coupling may make such determinations challenging for other experimental methods.

In the realm of X-ray spectroscopic studies, XES has become a viable tool to monitor spin state and/or oxidation states in both static samples and in situ or operando studies. Some of the clearest utility of Fe Kβ XES has been to discriminate between low- versus high-spin iron species [56–60]. The lack of significant shifts in Kβ_1,3_ mainline energies for the different oxidation states of MMOH intermediates precludes one from attempting more advanced X-ray spectroscopic techniques, such as “site selective” XAS or EXAFS for further enhancement of the MMOH_Q_ signal in the mixed sample through the means of energy selective detection [76, 77]. The mainline spectra reported here will be valuable in future studies of sMMO. With the previous success of using Mn Kβ emission to fingerprint oxidation state during in situ measurements [70, 78], one hopes to be able to utilize the Fe Kβ XES to fingerprint, monitor and validate electronic changes during in situ studies. Such XES approaches have been highlighted in X-ray free electron laser (XFEL) protein diffraction studies to monitor a catalytic site’s oxidation-state [79, 80]. The present results on the Fe Kβ mainlines of sMMO, however, demonstrate the potential difficulty of employing such methods for monitoring oxidation-state during in situ studies, where the Kβ_1,3_ energy does not offer great sensitivity but the Kβ’ itself offers spin state (correlated to the oxidation-state) information through its clear intensity changes. Given the inherently lower intensity of the Kβ’ feature, using this feature to follow spin state will require data collection with high signal-to-noise to ensure accurate assignments. We do note that the Kα (2p → 1 s) emission spectrum is an order of magnitude more intense than the Kβ emission, allowing for easier measurements. Kα XES does not offer the same degree of electronic sensitivity, and extraction of oxidation or spin-state information is challenging [54, 58]. The three states of sMMO do not exhibit significant energy differences in their Kα spectra (Figure S6 in ref [24].) and more recent Kα XES of MMOH_red_ and MMOH_ox_ collected during XFEL crystallography experiments exhibits only subtle changes in the linewidths and shapes [81]. While more challenging to collect, Kβ XES appears to offer the most sensitivity to spin state and the best potential fingerprint for formation of the di-iron(IV) (*S*_loc_ = 2) cluster.

Fe Kβ XES of sMMO has proven to be a valuable technique to probe the local spin states of the di-iron site in the catalytic cluster. Understanding and verifying that MMOH_Q_ is, in fact, *S*_loc_ = 2 is important to both understanding and proposing mechanisms for reactivity. Further, the ability to clearly distinguish *S* = 1 from *S* = 2 iron sites via Kβ XES has potential for experimentally assessing the role of two-state reactivity in a wide range of high-valent Fe^IV^–oxo complexes [48, 82]. Hence, the present results deepen our understanding of the electronic structure of MMOH_Q_, while providing experimental fingerprints to enable the study of a wide range of high-valent iron species in both molecular models and enzymes.

## Supplementary Information

Below is the link to the electronic supplementary material.

Supplementary file1 (PDF 426 kb)

## Data Availability

The datasets generated during and/or analyzed during the current study are available from the corresponding author on reasonable request.
