# Supplementary material for: Determination of the iron(IV) local spin states of the Q intermediate of soluble methane monooxygenase by Kβ X-ray emission spectroscopy
Source: J Biol Inorg Chem. 2022 Aug 21;27(6):573–82. doi: 10.1007/s00775-022-01953-4 (PMC9470658; doi:10.1007/s00775-022-01953-4)
Supplement: Supplementary file 1 — Supplementary file1 (PDF 426 kb) [file 775_2022_1953_MOESM1_ESM.pdf]

Supplemental Information

for

Determination of the Iron(IV) Local Spin-States of the Q Intermediate of Soluble Methane Monooxygenase by Kβ X-ray Emission Spectroscopy

George E. Cutsail III[1,2*] Rahul Banerjee,[3] Derek B. Rice,[1] Olivia McCubbin Stepanic,[1] John D. Lipscomb,[3] Serena DeBeer,[1*]

[1] Max Planck Institute for Chemical Energy Conversion, Stiftstrasse 34-36, D-45470 Mülheim an der Ruhr, Germany

[2] Institute of Inorganic Chemistry, University of Duisburg-Essen, Universitätsstrasse 5-7, D-45117 Essen, Germany

[3] Department of Biochemistry, Molecular Biology, and Biophysics, University of Minnesota, Minneapolis, Minnesota 55455, USA

email: george.cutsail@cec.mpg.de
email: serena.debeer@cec.mpg.de

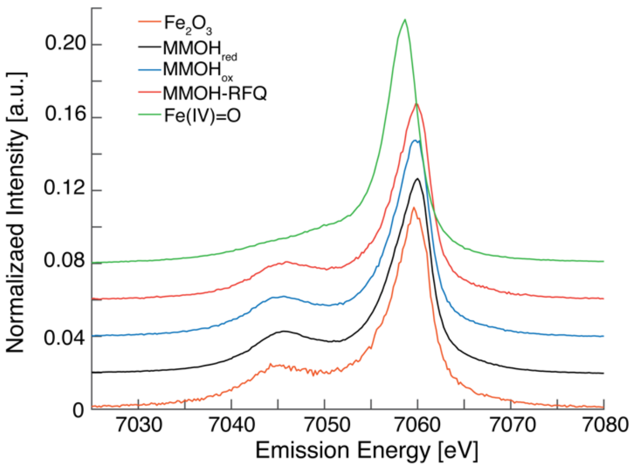

Figure S1. Reference Fe_2O_3 Fe Kβ XES spectrum together with all measured spectra from the current study.

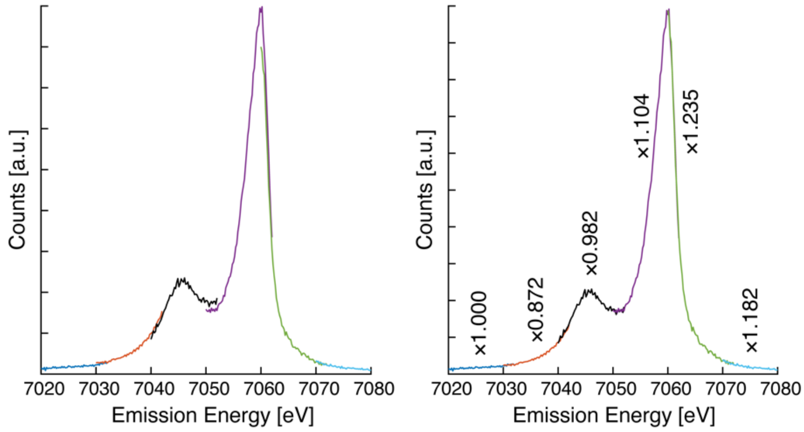

Figure S2. Sample of segmented XES collection pre- and post-splining. (left) As collected, incident beam normalized, XES signal of each data segment collected on a fresh sample spot. (right) Splined data by scaling each segment sequentially to the mean of the overlapping region of data collected. Scaled values for this data and each segment are given.

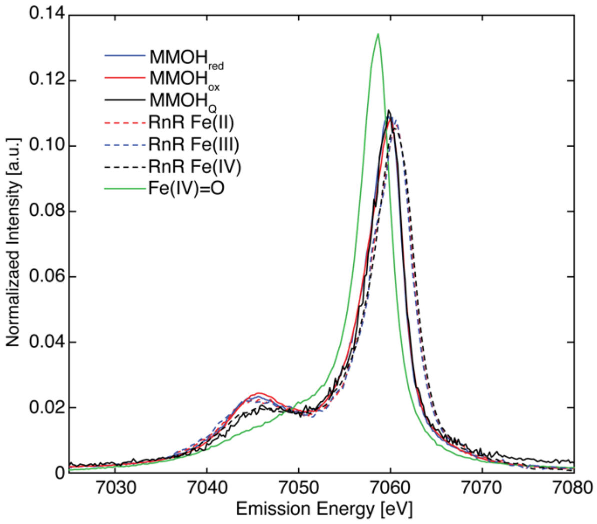

Figure S3. Fe Kβ mainlines of MMOH, MnFe RNR and Fe(IV)=O. The Fe XES of the heteronuclear MnFe RNR is of a single high-spin (S = 2) Fe(IV) site. The RNR mainlines were digitized from Martinie et al.[1] and are shifted due to apparent differences in spectrometer calibrations.

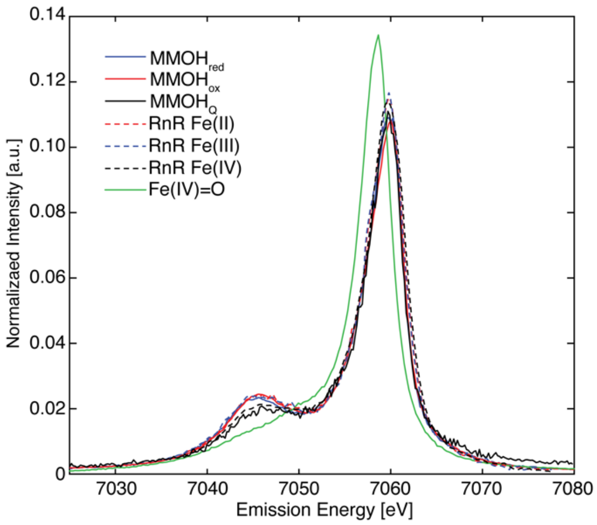

Figure S4. Fe Kβ mainlines as shown in Figure S3, except the energy scale of the RNR Fe mainlines has been compressed by the following equation: En(sMMO) = En(RNR)*.925 +528.75, where En(sMMO) is the final energy sMMO XES dataset and En(RNR) was the energy of the digitized RNR data from Martinie et al.[1]. The energy scale of the RNR data was effectively compressed so that the diferrous Fe Kβ mainline peak positions overlaid.

1 R. J. Martinie, E. J. Blaesi, J. M. Bollinger, Jr., C. Krebs, K. D. Finkelstein and C. J. Pollock (2018) Angew Chem Int Ed 57:12754-12758 https://doi.org/10.1002/anie.201807366
